# Fluorescence to highlight the urethra: a human cadaveric study

**DOI:** 10.1007/s10151-017-1615-y

**Published:** 2017-05-30

**Authors:** T. G. Barnes, M. Penna, R. Hompes, C. Cunningham

**Affiliations:** 1 Department of Colorectal Surgery, Oxford University Hospitals NHS Foundation Trust, Oxford, UK; 2 Nuffield Department of Surgery, John Radcliffe Hospital, Headley Way, Headington, OX3 9DS UK

**Keywords:** Colorectal surgery, Laparoscopic surgery, Fluorescence, Urethral injury, Urethra, Rectal cancer

## Abstract

**Background:**

Urethral injury is a complication feared by surgeons performing transanal TME (TaTME) or abdominoperineal excision (APE) procedures. Injury during TaTME occurs when the prostate is inadvertently mobilised or as a direct injury similar to the direct injury during the perineal dissection of APE procedures. We performed a proof of principle study to assess the feasibility of using indocyanine green (ICG) to fluoresce the urethra in human cadavers.

**Methods:**

Indocyanine green at varying doses was mixed with Instillagel and infiltrated into the urethra of male human cadavers. The urethra was exposed through either a perineal incision or by mobilisation of the prostate during a TaTME dissection and fluorescence observed using a PINPOINT laparoscope (NOVADAQ). Brightness was assessed on the images using ImageJ (National Institute of Health).

**Results:**

Eight cadavers were included in the study. Fluorescence was visualised in the urethra in all eight cadavers. Minimal dissection was required to obtain fluorescence transperineally. In one cadaver, the urethra was demonstrated under fluorescence using a simulated TaTME with additional fluorescence also being observed in the prostate. There was no correlation between brightness and dosing.

**Conclusions:**

This novel proof of principle study demonstrates a simple way in which the urethra may be easily identified preventing it from injury during surgery.

**Electronic supplementary material:**

The online version of this article (doi:10.1007/s10151-017-1615-y) contains supplementary material, which is available to authorized users.

## Introduction

Operative management of rectal cancer involves anterior resection in the form of a total mesorectal excision (TME) or, for low rectal cancers invading the sphincter muscles, abdominoperineal excision (APE) of the rectum is appropriate. Fifty-five per cent of patients diagnosed with rectal cancer undergo surgical resection, amounting to 5000 patients per year with 35% of these patients having neoadjuvant radiotherapy [1].

A recently developed procedure for TME dissection is to perform the procedure via a combined laparoscopic abdominal and endoscopic approach: transanal total mesorectal excision (TaTME). One major concern that has arisen regarding TaTME procedures is the risk of urethral injury [2, 3]. As many as 24 documented urethral injuries have occurred during the clinical adoption of this new technique (personal communication, Professor Patricia Sylla MD, Mount Sinai Hospital, New York). The injury occurs following inadvertent mobilisation of the prostate, putting the membranous urethra at risk [4]. This injury may be avoided with adequate training and mentoring in the technique. However, direct injury can still occur without prostate mobilisation during perineal dissection in an intersphincteric approach or APE.

Dissection in the plane anterior to the anus is an area of concern even for experienced cancer surgeons, aggravated by unfavourable anatomy and the presence of an anterior rectal cancer. Increasing use of video-directed rectal cancer surgery [5] means that it is not often possible to palpate the urethral catheter during perineal dissection; therefore, enhanced reality imaging may be of value in defining the anatomy. The incidence of urethral injury during colorectal surgery is around 3% [6] and is more commonly seen in patients who have had neoadjuvant radiotherapy. The post-operative sequelae are a source of significant morbidity and frustration for the patient [7].

This study was a proof of principle study investigating the use of indocyanine green (ICG) as a fluorophore to help identify the urethra during all types of perineal dissection where the urethra may be difficult to visualise.

## Materials and methods

Eight freshly frozen male cadavers were obtained through the Department of Anatomy, University of Oxford, and the study was ethically approved internally by the same department. All cadavers had been used for a TaTME training course earlier the same day. Cadavers were placed in a modified Lloyd Davies position and where possible, a 14fr Foley catheter was placed in the urethra with 10 ml of saline used to inflate the balloon.

ICG (Verdye, Diagnostic Green, Aschheim-Dornach, Germany) was reconstituted using normal saline and mixed with 10 ml of Instillagel (CliniMed, Loudwater, High Wycombe, Bucks, UK). Ten millilitres of ICG was infiltrated directly into the lumen of the urethra whilst the urethra was manually occluded. Where a urinary catheter was in place, ICG was infiltrated adjacent to the catheter into the urethra. Dosing of ICG was as follows: 2.5, 5, 10, 12.5 and 25 mg. Dissection was performed down to the urethral wall with observation of fluorescence signal using the PINPOINT laparoscopic system (NOVADAQ, Bonita Springs, FL, USA). The PINPOINT system emits a laser at 806 nm and filters the fluorescence signal at 835 nm.

The urethra was then exposed through the perineum using either a transverse or vertical incision. In cadavers where the prostate had been intentionally mobilised as part of the learning process during the TaTME procedure, the anastomosis was taken down, the abdomen closed and the urethra visualised through the transanal platform.

Brightness values were calculated using ImageJ (version 2.0.0) [8]. A freehand selection was drawn around the urethra in white light mode and applied to the images obtained under fluorescence and brightness measure (the software calculates brightness values by calculating the mean of red, green and blue pixels). Data were analysed using Prism (V7.0a, Graphpad) and SPSS statistics (version 22.0, IBM corp). Where there was more than one cadaver for a dosing cohort, the means and standard deviations were combined using a standard formula [9]. The relationship between dosing and brightness was calculated using linear regression.

## Results

All cadavers displayed fluorescence in the urethra after infiltration of ICG/Instillagel mixture. A perineal incision was utilised in seven cadavers. Of these, five had a urinary catheter in situ and we subjectively observed similar levels of fluorescence to those in cadavers without a urinary catheter. To assess whether ICG could be infiltrated down a lumen of a Foley catheter rather than the urethra, we imaged a catheter using ICG to the fill balloon lumen. Using the PINPOINT laparoscope, no fluorescence was observed along the any part of the length of the catheter.

**Fig. 1:**
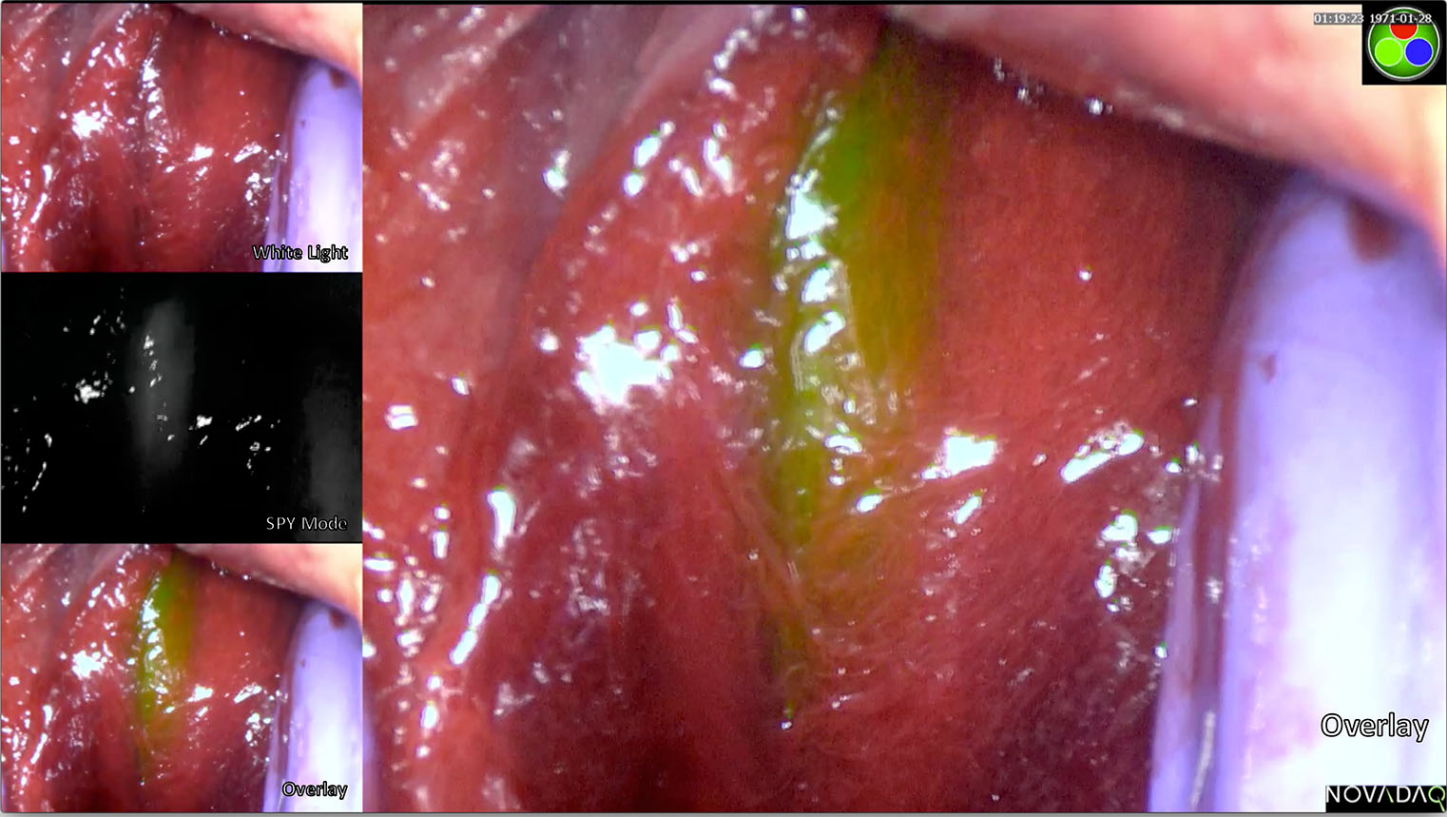
Fluorescence clearly seen in both SPY mode and the overlay. In the white light image (top left thumbnail), the urethra is not seen with white light alone

**Fig. 2:**
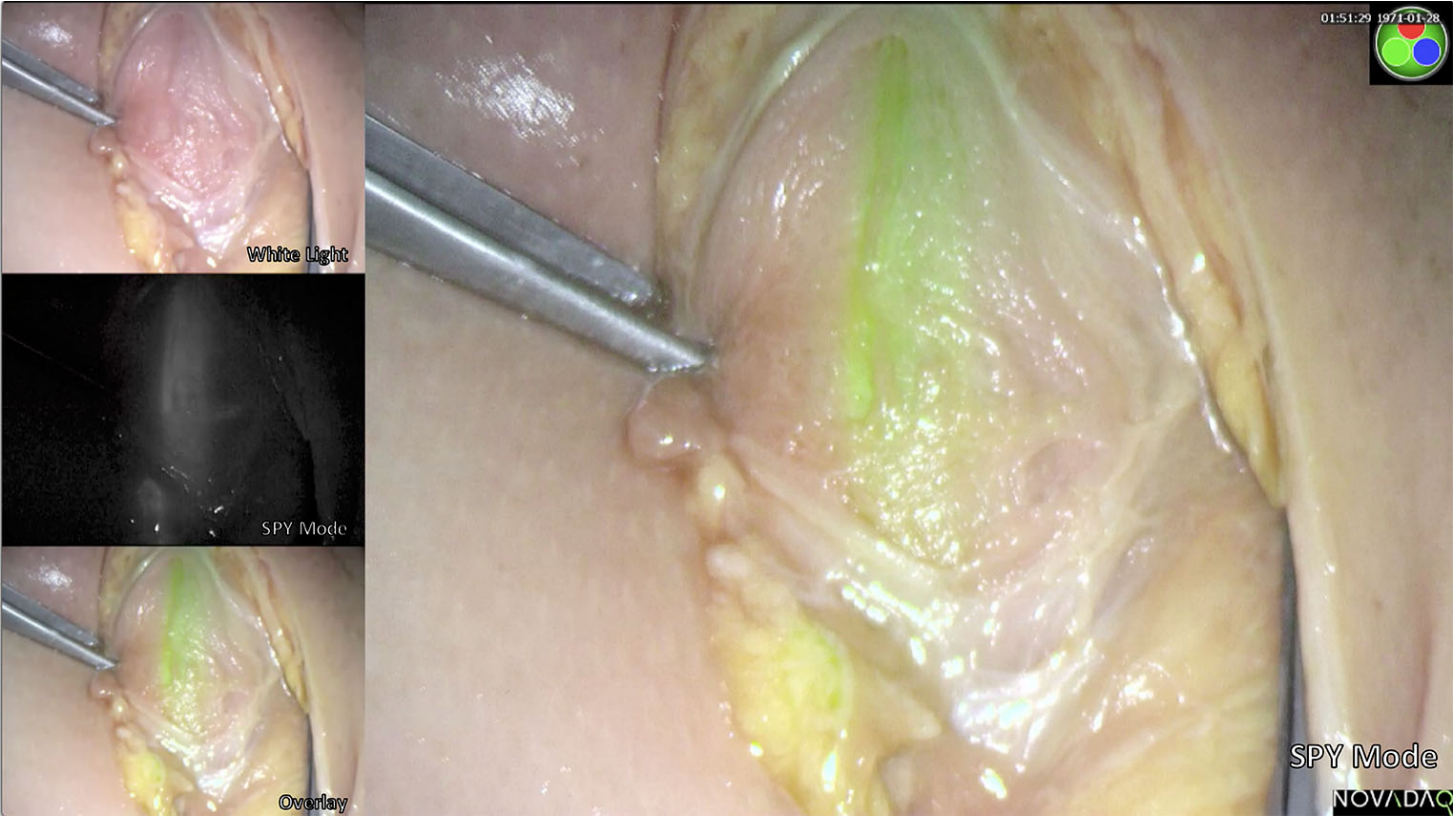
Urethral dissection without entering the overlying muscle and fluorescence is still seen—SPY mode

The PINPOINT system allows visualisation of pure fluorescent signal as a black and white image (SPY mode) and an overlay of fluorescence (green) on a white light image. Example images of urethral fluorescence are shown in Figs. 1 and 2.

**Fig. 3:**
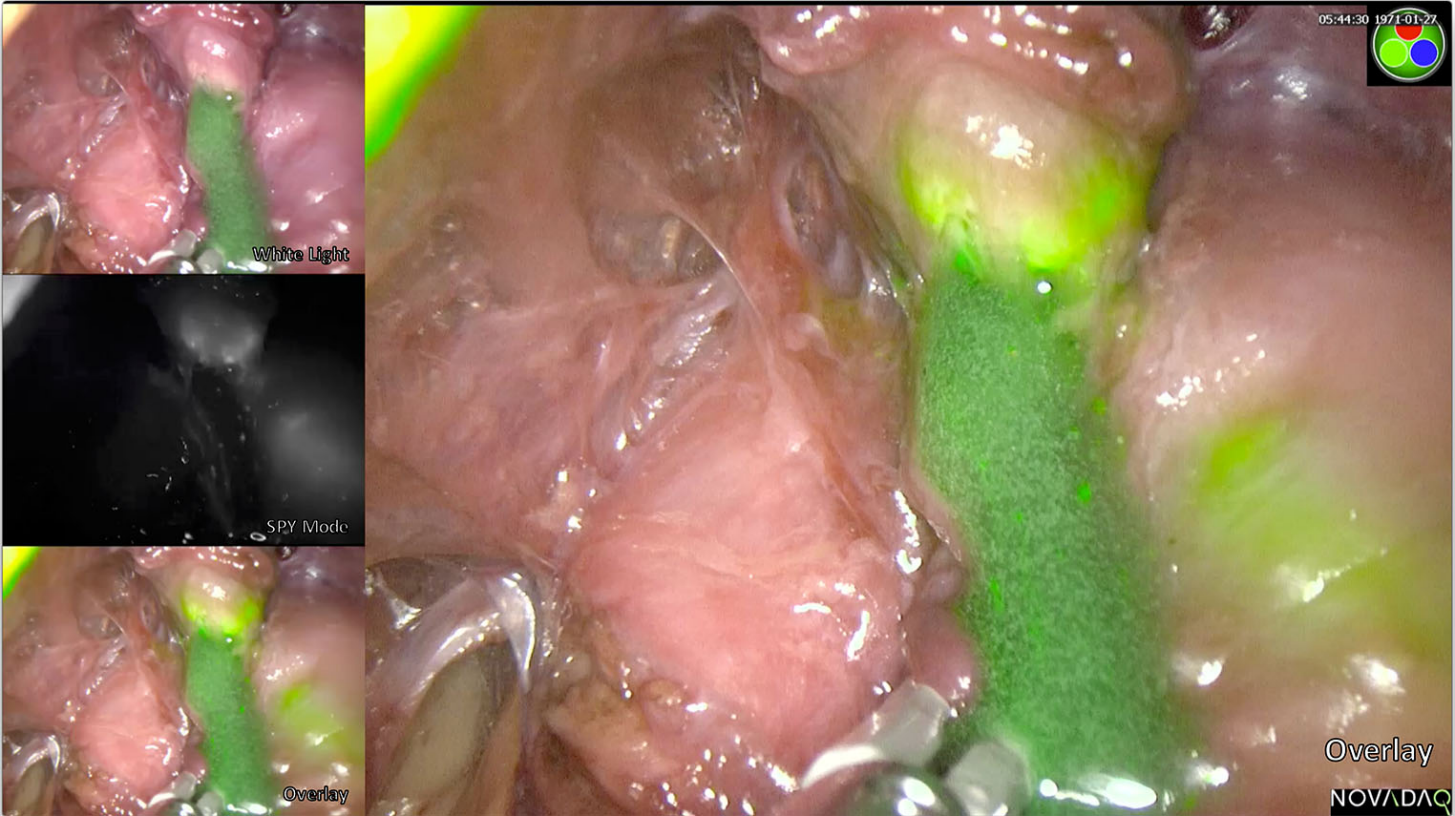
On breaching the urethra, there is a leak of the Instillagel containing ICG. Whilst this appears green on a white light image, there is no true fluorescence (SPY mode) unless ICG is bound to the tissue

It was not necessary to completely denude the urethra in any of the cadavers as fluorescence was seen through the overlying muscle. After exposing the urethra and observing fluorescence, we deliberately breached the urethral wall and observed leakage of ICG and Instillagel. As expected, the leaking material did not fluoresce but was green under white light (Fig. 3).

**Fig. 4:**
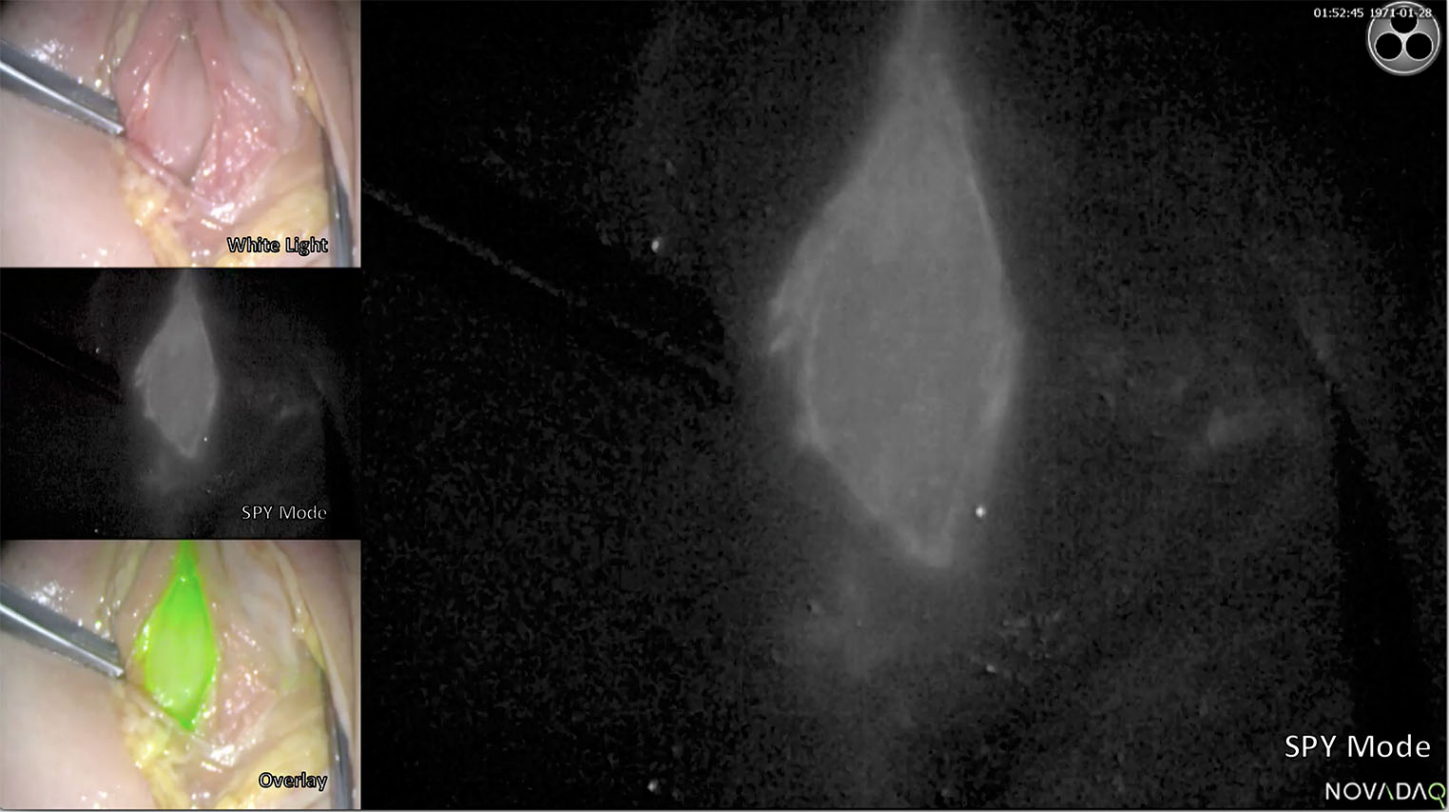
After breaching the urethra, the ICG is seen to have stained the lining of the urethra

In the cadavers, where it was not possible to insert a catheter, there was concern about ICG leaking into the bladder and the fluorescence being lost. The opposite occurred and ICG appeared to ‘stain’ the lining of the urethra (Fig. 4).

**Fig. 5:**
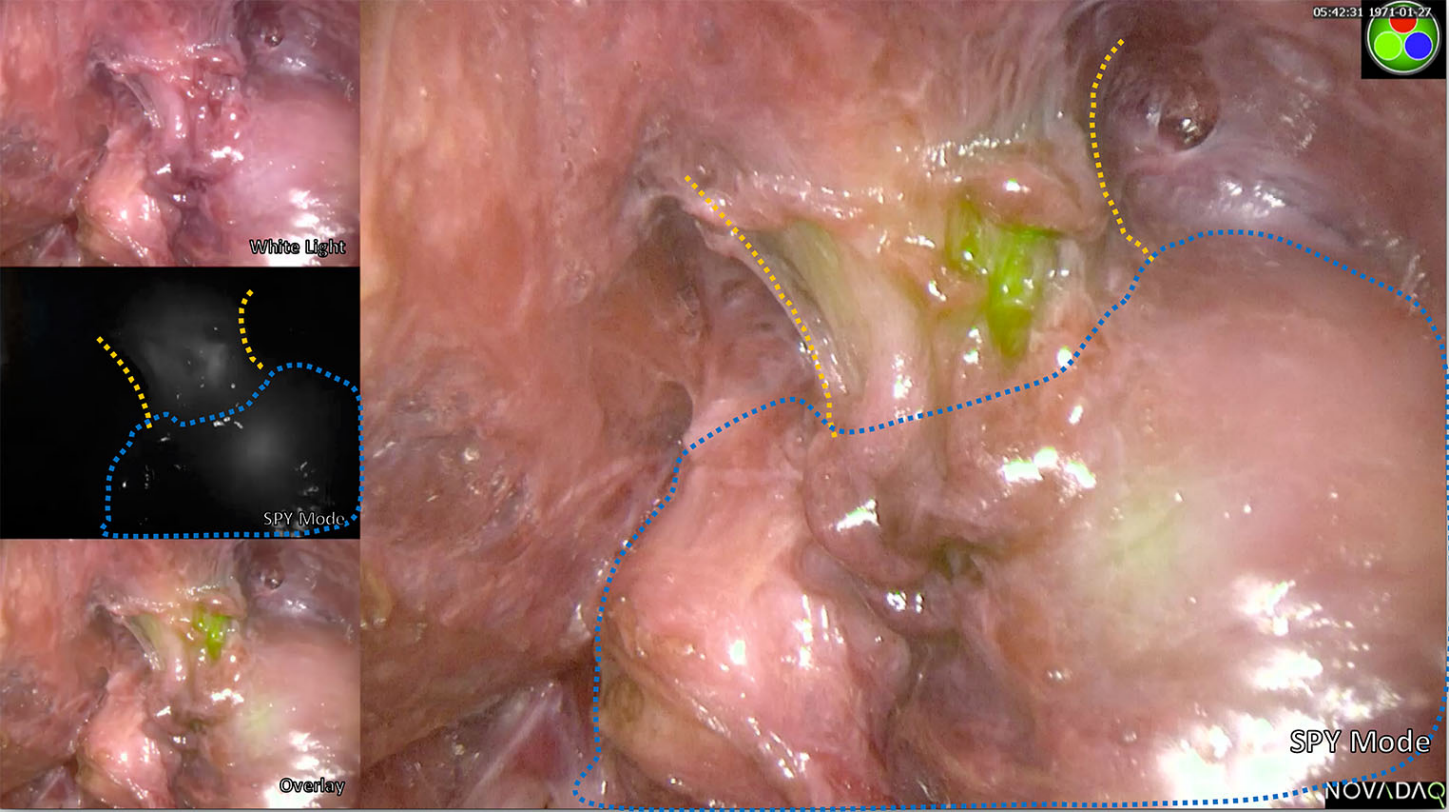
Demonstration of urethral fluorescence during a simulated TaTME. *Orange line* urethral outline; *Blue outline* prostate

In one cadaver, the prostate had been inadvertently mobilised during the TaTME dissection. With the transanal platform in situ, ICG was infiltrated and fluorescence was observed clearly delineating the urethra. The prostate was further mobilised purposely, and signal was also detected in the prostate (Fig. 5).

**Fig. 6:**
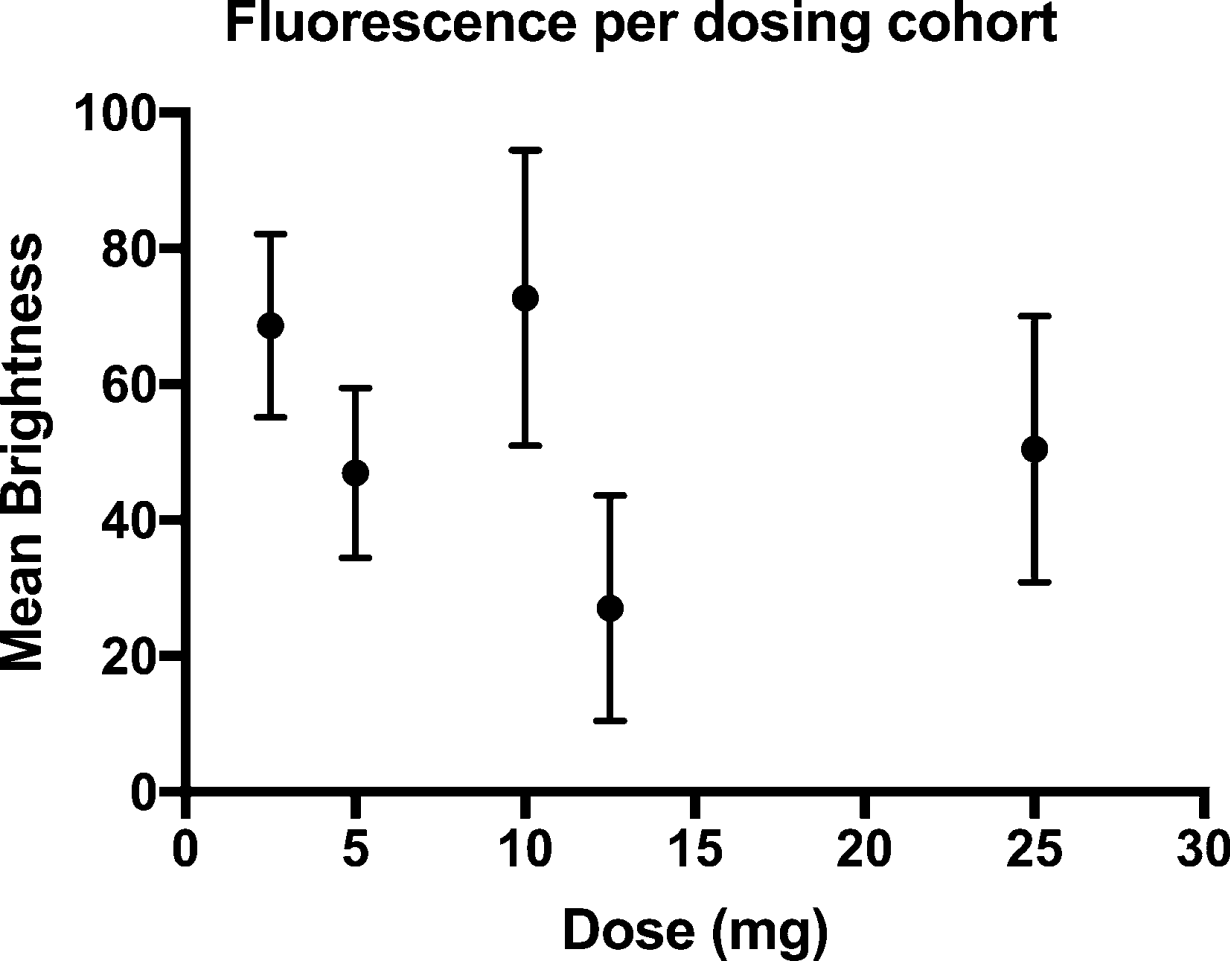
Relationship of brightness against dose of ICG. *Dots* represent the mean or combined mean brightness for each dose and *error bars* standard deviation

Comparison of brightness between dosing cohorts revealed no relationship between dose and brightness (Pearson’s correlation coefficient −0.297, *p* = 0.314) (Fig. 6).

## Discussion

This proof of principle study demonstrates that there is a potential use for fluorescence in identifying and protecting the urethra during colorectal surgery. This could be applied to TaTME, intersphincteric dissections or APE procedures, where the urethra is particularly at risk.

In all eight cadavers, fluorescence signal was observed in the urethra after infiltration. The urethra was easily seen through a perineal incision and demonstrates that the ICG emission easily penetrates the overlying muscle to allow visualisation to a depth of 2–4 cm [10, 11]. ICG is known to have a low level of fluorescence in aqueous solution with its emission increasing when bound to plasma proteins or cellular membranes [12]. This explains the observation that fluorescence was observed ‘sticking’ to the mucous membrane of the urethra and not observed in the leaked Instillagel. ICG is commonly used intravenously to assess perfusion and subcutaneously, submucosally or subserosally to assess lymphatics. There is no published literature surrounding its application directly onto mucous membranes [13] nor is it licensed for this purpose. Future work would require a feasibility study to assess dosing, timing and safety for its use intraoperatively. It is likely to be a safe technique as we demonstrated that a low dose (2.5 mg) can be utilised with the likelihood of lower doses being efficacious. Infiltration of the catheter with ICG rather than the urethra does not produce detectable fluorescence.

This technique is likely to be of greatest benefit in APE procedures. This technology can potentially enhance TaTME, and reassurance would be provided where fluorescence is not visualised. Whilst one would not expect to see the urethra when in the correct plane during TaTME, the ability of urethral fluorescence to also highlight the prostate has been demonstrated here and may even help the surgeon to determine the correct plane during the anterior dissection.

## Conclusions

This novel technique using fluorescence may help prevent urethral injury during perineal dissection. Studies are needed to establish its feasibility and safety in live patients.

## Electronic supplementary material

Below is the link to the electronic supplementary material.

Supplementary material 1 (MP4 329836 kb)
